# Gross anatomical features of the insular cortex in affective disorders

**DOI:** 10.3389/fpsyt.2024.1482990

**Published:** 2024-12-09

**Authors:** Tsutomu Takahashi, Daiki Sasabayashi, Murat Yücel, Sarah Whittle, Michio Suzuki, Christos Pantelis, Gin S. Malhi, Nicholas B. Allen

**Affiliations:** ^1^ Department of Neuropsychiatry, University of Toyama School of Medicine and Pharmaceutical Sciences, Toyama, Japan; ^2^ Research Center for Idling Brain Science, University of Toyama, Toyama, Japan; ^3^ QIMR Berghofer Medical Research Institute, Brisbane, QLD, Australia; ^4^ Melbourne Neuropsychiatry Centre, Department of Psychiatry, The University of Melbourne and Melbourne Health, Melbourne, VIC, Australia; ^5^ Florey Institute of Neuroscience and Mental Health, University of Melbourne, Melbourne, VIC, Australia; ^6^ North Western Mental Health, Western Hospital Sunshine, St Albans, VIC, Australia; ^7^ 7Academic Department of Psychiatry, Kolling Institute, Northern Clinical School, Faculty of Medicine and Health, The University of Sydney, Sydney, NSW, Australia; ^8^ CADE Clinic, Royal North Shore Hospital, Northern Sydney Local Health District, Sydney, NSW, Australia; ^9^ Department of Psychiatry, University of Oxford, Oxford, United Kingdom; ^10^ Department of Psychology, University of Oregon, Eugene, OR, United States

**Keywords:** magnetic resonance imaging, insular cortex, neurodevelopment, major depressive disorder, bipolar disorder

## Abstract

**Introduction:**

The number of insular gyri is elevated in patients with schizophrenia. Thus, it has potential as a marker of early neurodevelopmental abnormalities. However, currently it remains unclear whether patients with other neuropsychiatric disorders, such as affective disorders, also have this gross brain anatomical feature.

**Materials and methods:**

The macroscopic features of the insular cortex in 26 patients with bipolar disorder (BD), 56 with major depressive disorder (MDD), and control subjects for each clinical group (24 for BD and 33 for MDD) were assessed using magnetic resonance imaging.

**Results:**

The number of short insular gyri was higher in BD patients than in matched controls bilaterally with well-developed accessory and middle short gyri. Furthermore, the left middle short gyrus was more developed in MDD patients than in matched controls, and was weakly associated with the severity of depressive symptoms.

**Discussion:**

The present results indicate that changes in the gross morphology of the insular cortex in BD and MDD is a potential vulnerability factor associated with their neurodevelopmental pathologies, and may also contribute to the severity of symptoms in MDD.

## Introduction

The insula, particularly its anterior portion (i.e., the short insula), plays a crucial role in socio-emotional processing within the context of the limbic integrating cortex (1, 2), and changes in its structure (3, 4) and function (5, 6) are thought to contribute to the pathophysiological processes underlying affective disorders. Large inter-individual variability has been reported in gyrification patterns in the insula, which potentially reflect cytoarchitectonic development during gestation (7–9). The accessory gyrus (AG) and middle short gyrus (MSG) in the short insula are often undeveloped or absent in human brains (up to 50-70%), while the posterior long gyrus (PLG) in the posterior subdivision (i.e., long insula) is missing in approximately 10-20% (10–12). It has not yet been established whether these macroscopic features of the insular cortex affect brain function, whereas deviations in gross brain characteristics, particularly gyrification patterns, have been suggested to reflect the neurodevelopmental pathologies associated with various neuropsychiatric disorders (13).

We previously demonstrated, using magnetic resonance imaging (MRI), that the number of insular gyri was elevated in patients with schizophrenia regardless of the illness stage, indicating its potential as a neurodevelopmental marker (14–16); however, the disease specificity of this finding remains unknown. Although the neurological basis of affective disorders has not yet been elucidated in detail, developmentally mediated neurobiological changes related to socio-emotional neural circuits may contribute to their pathophysiologies, particularly that of bipolar disorder (BD) (17, 18). Major depressive disorder (MDD) is a phenotypically heterogeneous disorder that is caused by a combination of biological and environmental factors (19, 20), while embryonic neurodevelopmental abnormalities have also been suggested to contribute to the later development of MDD (21, 22). We previously revealed similar changes in the brain surface morphologies of schizophrenia (23), BD (24), and MDD (25), indicating a partial overlap in their neurodevelopmental pathologies (26). Despite the potential role of insular abnormalities in affective disorders (27, 28), the gross anatomy of the insular cortex has not yet been investigated in detail in BD and MDD.

Therefore, in the present study, MRI was performed on patients with BD, those with MDD, and matched control subjects to examine the macroscopic features of the insular cortex (i.e., the number of gyri and degree of development of each gyrus). Due to the potential contribution of insular abnormalities to emotional dysregulation in affective disorders (27, 28) and neurobiological overlap with schizophrenia in terms of gross brain anatomy (26), we hypothesized that the number of insular gyri with well-developed gyri may be higher in patients with affective disorders (particularly BD) than in healthy controls. Furthermore, we investigated the relationship between the anatomy of the insular cortex and the clinical features of these patients (e.g., symptoms and medication).

## Materials and methods

### Participants

Twenty-six patients with BD, 56 with MDD, and 57 healthy controls who were right-handed and had no previous history of serious head trauma, neurological illness, substance misuse, or other serious physical diseases were enrolled in the present study (**Table 1**). The sample characteristics of these participants and inclusion/exclusion criteria were reported in detail in previous studies (24, 25, 29, 30).

**Table 1:** Characteristics of study participants.

	BD cohort		MDD cohort	
	Patients (*N* = 26)	Controls (*N* = 24)	Patients (*N* = 56)	Controls (*N* = 33)
Age (years)	38.4 ± 10.9	38.7 ± 11.1	33.8 ± 9.1	34.0 ± 9.9
Male/female	8/18	7/17	16/40	12/21
Current IQ	113.8 ± 7.1	115.1 ± 9.6	108.0 ± 9.8	111.1 ± 10.9
Onset age (years)	24.9 ± 8.4	–	23.5 ± 9.0	–
Duration of illness (years)	13.5 ± 10.1	–	10.3 ± 8.1	–
Number of depressive episodes	11.1 ± 10.8	–	3.4 ± 3.0	–
Number of manic episodes	8.8 ± 10.2	–	–	–
Beck Depression Inventory	–	–	23.4 ± 15.8	3.6 ± 4.1
PANAS positive affect	–	–	25.0 ± 8.0	32.9 ± 7.3
PANAS negative affect	–	–	17.8 ± 7.7	11.2 ± 1.6
MASQ general distress	–	–	45.8 ± 10.3	27.9 ± 8.3
MASQ general depression	–	–	41.5 ± 12.0	19.5 ± 7.2
MASQ general anxiety	–	–	28.7 ± 9.0	16.4 ± 6.4
MASQ anxious arousal	–	–	36.1 ± 12.2	22.0 ± 4.4
MASQ high positive affect	–	–	53.5 ± 16.8	81.1 ± 14.3
MASQ loss of interest	–	–	27.8 ± 7.7	14.7 ± 5.0
Medication at scanning [*N* (%)]	21/26 (80.8%)	–	33/56 (58.9%)	–

Values represent means ± SD unless otherwise stated. BD, bipolar disorder; MASQ, Mood and Anxiety Symptom Questionnaire; MDD, major depressive disorder; PANAS, Positive and Negative Affect Schedule.

Briefly, we recruited BD patients from the Mood Disorders Unit at the Prince of Wales Hospital, Sydney, Australia. They had been diagnosed with bipolar I disorder according to the Structured Clinical Interview for DSM-IV patient version (SCID-IV-P) by research psychiatrists (31). We also performed a detailed case note review of the clinical characteristics of these patients, including the lifetime number of affective episodes and their medication status. A history of psychotic symptoms (i.e., delusions and/or hallucinations) during previous affective episodes was noted in 16 BD patients and a family history of affective disorders in 10. At the time of scanning, all patients were in a euthymic condition (i.e., not currently experiencing a manic/hypomanic or depressive episode of SCID). All patients had previously been administered antipsychotics, but not within 1 year of participating in the present study. At the time of the present study, 19 patients were being treated with mood stabilizers, including lithium (Li) (*N* = 12) and/or valproate (VPA) (*N* = 12).

Patients with MDD were enrolled from outpatient psychiatric clinics or an advertisement in the local media in Melbourne, Australia. They had been diagnosed with MDD according to the SCID-IV-P by experienced research psychologists at Orygen Youth Health, Melbourne (31), and had also been assessed using the Beck Depression Inventory (BDI) (32), Positive Affect and Negative Affect Scale (PANAS) (33), and Mood and Anxiety Symptom Questionnaire (MASQ) (34). The case history, medication status, and comorbid anxiety disorders of these patients were also examined through a direct interview and chart review. At the time of scanning, there were 27 patients in remission (remitted MDD subgroup) and 29 who fulfilled the DSM criteria for MDD (currently depressed subgroup). MDD was comorbid with anxiety disorders in 22 (4 remitted and 18 currently depressed) patients.

Healthy controls matched on age and sex were recruited in Sydney (*N* = 24, matched for BD) and Melbourne (*N* = 33, matched for MDD) through a local advertisement. They also underwent screening using the SCID-IV non-patient version (31) and none had a personal or family history of psychiatric diseases. The Melbourne Health Mental Health Research & Ethics Committee approved the present study (MHREC2009.607). Based on the tenets of the Declaration of Helsinki, all participants provided their written informed consent after receiving a complete description of the study protocol.

### MRI procedure

Details of the MRI procedures performed on the Sydney BD and Melbourne MDD cohorts have been described in previous studies (24, 25, 29). Briefly, fast-spoiled gradient echo MR imaging using the 1.5-T GE Signa scanner at Royal Prince Alfred Hospital in Sydney was performed to create T1-weighted consecutive coronal images with a voxel size of 0.98 × 0.98 × 1.6 mm for the BD cohort and their matched controls. The 1.5T Siemens scanner (Magnetom Avanto) at Saint Vincent’s Hospital Melbourne was used to obtain T1-weighted 1-mm iso-voxel images in the axial orientation for the MDD cohort and their controls.

MR images, including information on diagnoses, were fully anonymized and were reconstructed into 0.98-mm (BD)- or 1-mm (MDD)-thick entire contiguous coronal images perpendicular to the anterior commissure-posterior commissure line using Dr. View software (Infocom, Tokyo, Japan).

### Insular gross anatomy assessment

**Figure 1:**
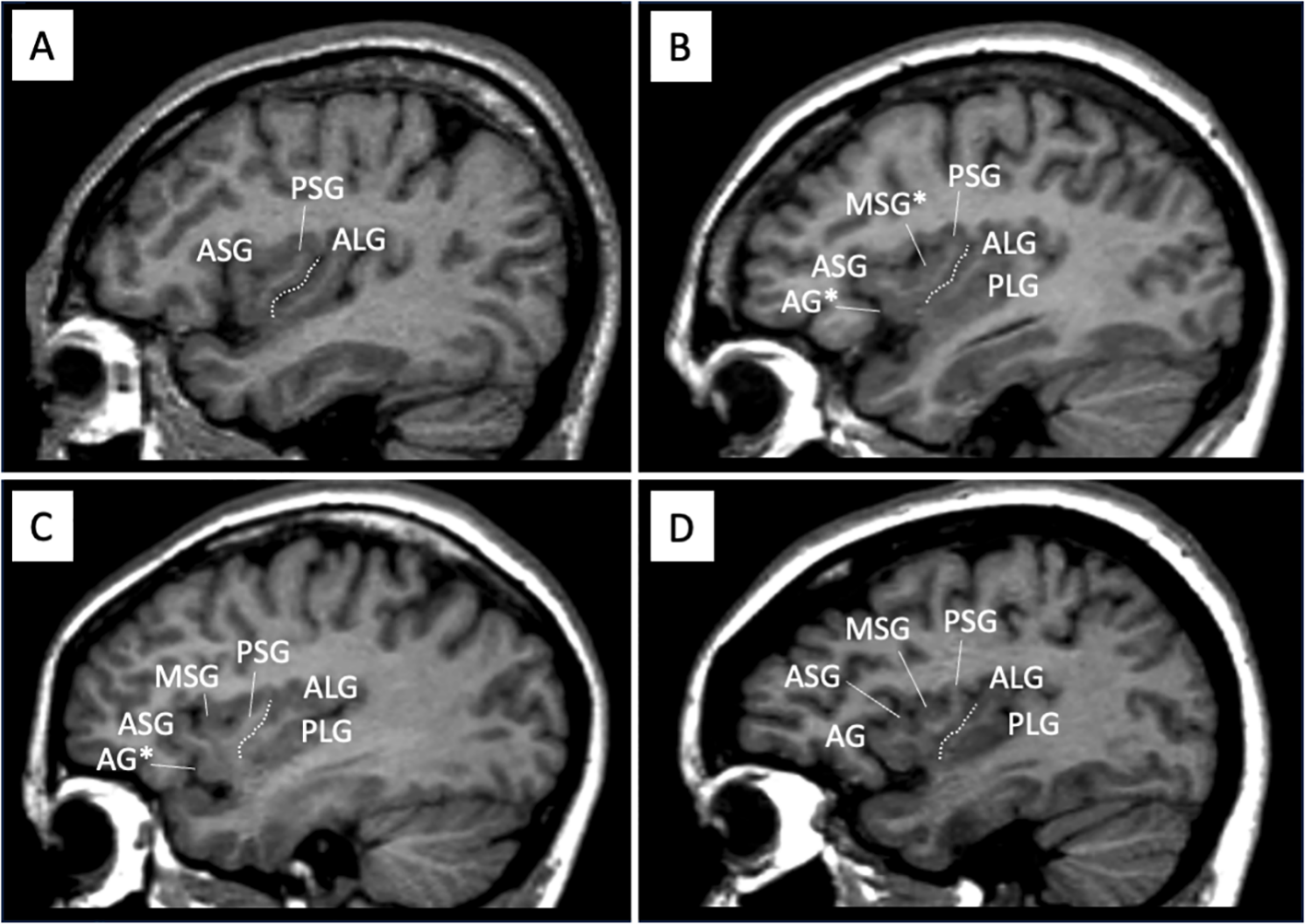
Sample sagittal MR images of different gross insular patterns. The developmental pattern of each gyrus was assessed predominantly using consecutive sagittal slices, with also coronal and axial views simultaneously being referred to. Dotted lines indicate the location of the central insular sulcus that subdivides the insula into the anterior (short) and posterior (long) cortices. The ASG, PSG, and ALG were well-developed in all participants in this study, while the PLS was sometimes deficient panel **(A)**. The AG and MSG in the short insula had a large inter-individual variation, where the gyrus was absent panel **(A)**, underdeveloped [marked with asterisks in panels **(B, C)**], or well-developed panel **(D)**. AG, accessory gyrus; ALG, anterior long gyrus; ASG, anterior short gyrus; MSG, middle short gyrus, PLS, posterior long gyrus; PSG, posterior short gyrus.

As described in detail in previous studies (14–16), AG, MSG, and PLG developmental patterns, which have substantial inter-individual anatomical variations (10–12), were evaluated by one rater (TT) (**Figure 1**). The classifications for AG and MSG were as follows: fully developed, underdeveloped, or absent, while that for PLG was present or absent because its hypoplasia is rarely observed in human brains (12). Only well-developed gyri were counted when assessing the number of anterior and posterior insular gyri.

A validity sample of 15 randomly selected brains (30 hemispheres) confirmed that intra-rater (TT) and inter-rater (TT and DS) reliabilities for the gyral number (intraclass correlation coefficients) and developmental pattern (Cronbach’s α) were both > 0.91.

### Statistical analysis

Demographic and clinical differences between groups were assessed using a one-way analysis of variance (ANOVA) or the χ^2^ test.

The χ^2^ test or Fisher’s exact test, when more than 20% of cells had expected counts < 5, was performed to examine group differences in AG, MSG, and PLG developmental patterns, where Benjamini-Hochberg procedure was used to decrease the false discovery rate. Since short and long insular gyri showed a non-normal distribution (Kolmogorov-Smirnov tests), their number was log-transformed and then compared between groups using an ANOVA, with the between-subject variables of diagnosis and sex and the within-subject factor of hemisphere. *Post-hoc* Scheffé’s test and Bonferroni correction were then conducted.

Two long insular gyri with a well-developed PLG were observed in the majority of the brains analyzed; therefore, the relationship between the gross anatomy of the insula and the clinical characteristics of patients was examined only for the short insular cortex. Spearman’s correlation analysis with the Bonferroni correction was employed to assess whether the number of short insular gyri was associated with clinical variables [IQ, onset age, illness duration, number of episodes, and symptom ratings (separately investigated for currently depressed and remitted MDD subgroups)]. The potential effects of the AG and MSG patterns (developed *vs.* underdeveloped or absent) on the clinical variables described above were also assessed using the Mann-Whitney U test because of the skewed distribution of most of these variables.

The relationship between the number of short insular gyri and clinical subgroups (currently depressed or remitted MDD subgroups, with or without comorbid anxiety disorders in the MDD group, with or without a history of psychosis or a family history of affective disorders in the BD group, and medication status) was investigated using the non-parametric Mann-Whitney U test.

Since we previously measured insular gray matter volumes in the Sydney BD (3) and Melbourne MDD (4) cohorts, Spearman’s correlation between the number of gyri and gray matter volume of the short insular cortex was examined in each hemisphere. The relationship between the AG and MSG patterns (developed *vs.* underdeveloped or absent) and gray matter volumes were also assessed using the Mann-Whitney U test. A *p* value < 0.05 indicated a significant difference.

## Results

### Demographic and clinical data

No significant differences were observed in age, sex, or IQ between BD and MDD patients and their controls (**Table 1**). Furthermore, these demographic variables did not significantly differ between the remitted and currently depressed MDD subgroups; however, depressive and anxiety symptoms were less severe and medication rates were lower in the remitted group than in the currently depressed group (25, 29).

### Variations in insular gross anatomy

**Figure 2:**
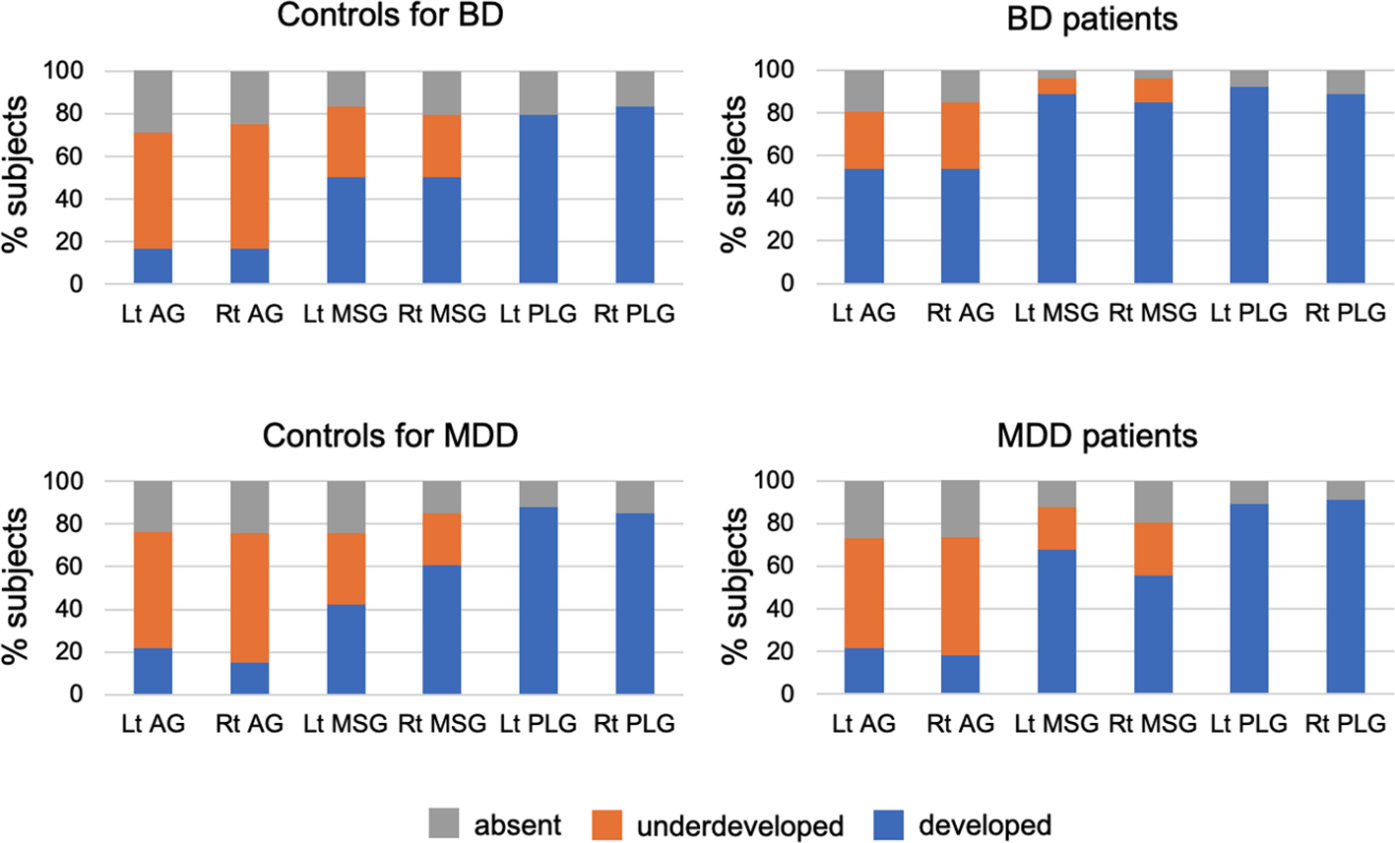
Developmental patterns of the accessory gyrus (AG), middle short gyrus (MSG), and posterior long gyrus (PLG) in the bipolar disorder (BD) group (*N* = 26), major depressive disorder (MDD) group (*N* = 56), and marched control groups for each patient group (24 subjects for BD and 33 for MDD). Variations in gyral development were not observed for other insular gyri (i.e., anterior short, posterior short, and anterior long gyri).

The number of bilateral short insular gyri was higher in BD patients than in controls matched to this group even after the Bonferroni correction [*p* < 0.0125 (0.05/4); BD/MDD groups by short/long gyri], with the bipolar group having a more well-developed AG (well-developed *vs.* underdeveloped or absent: left, χ^2^ = 7.49, *p* = 0.006; right, χ^2^ = 7.49, *p* = 0.006) and MSG (well-developed *vs.* underdeveloped or absent: left, χ^2^ = 8.79, *p* = 0.003; right, χ^2^ = 6.87, *p* = 0.009) (**Table 2**, **Figure 2**). The result of left MSG described above remained significant even after applying Benjamini-Hochberg procedure [*p* < 0.0042 (0.05/12); BD/MDD groups by left/right by AG/MSG/PLG]. No significant difference was noted in the gross insular anatomy of the long insular cortex between the groups.

**Table 2:** Gross anatomy of the insular cortex in study participants.

	BD cohort			MDD cohort		
	Patients (*N* = 26)	Controls (*N* = 24)	Group differences	Patients (*N* = 56)	Controls (*N* = 33)	Group differences
Number of short gyri			*F* (1, 46) = 19.97, *p* < 0.001^a)^			*F* (1, 85) = 0.01, *p* = 0.912^a)^
Left	3.42 ± 0.64	2.71 ± 0.69		2.91 ± 0.72	2.73 ± 0.67	
Right	3.46 ± 0.76	2.79 ± 0.72		2.75 ± 0.69	2.82 ± 0.68	
Number of long gyri			*F* (1,46) = 2.37, *p* = 0.131^a)^			*F* (1,85) = 1.02, *p* = 0.315 ^a)^
Left	1.92 ± 0.27	1.79 ± 0.41		1.89 ± 0.31	1.88 ± 0.33	
Right	1.88 ± 0.33	1.83 ± 0.38		1.91 ± 0.29	1.85 ± 0.36	
AG (developed/underdeveloped/absent)						
Left	14/7/5	4/13/7	Chi-squared = 7.62, *p* = 0.022	12/29/15	7/18/8	Chi-squared = 0.08, *p* = 0.960
Right	14/8/4	4/14/6	Chi-squared = 7.52, *p* = 0.023	10/31/15	5/20/8	Chi-squared = 0.24, *p* = 0.886
MSG (developed/underdeveloped/absent)						
Left	23/2/1	12/8/4	Fisher’s exact test, *p* = 0.010	38/11/7	14/11/8	Chi-squared = 5.57, *p* = 0.062
Right	22/3/1	12/7/5	Fisher’s exact test, *p* = 0.035	31/14/11	20/8/5	Chi-squared = 0.34, *p* = 0.845
PLG (developed/absent)						
Left	24/2	19/5	Fisher’s exact test, *p* = 0.239	50/6	29/4	Fisher’s exact test, *p* = 1.000
Right	23/3	20/4	Fisher’s exact test, *p* = 1.000	51/5	28/5	Chi-squared = 0.81, *p* = 0.369

Values represent means ± SD unless otherwise stated. AG, accessory gyrus; BD, bipolar disorder; MSG, middle short gyrus; MDD, major depressive disorder; PLG, posterior long gyrus. Statistical results with significant group differences of AG and MSG based on “well-developed *vs*. underdeveloped or absent” comparison were shown in the main text.

^a)^No sex or hemisphere main effects or their interactions were found.

Although the number of insular gyri did not significantly differ between MDD patients and their matched controls (**Table 2**), when the two classifications were compared (well-developed *vs.* underdeveloped or absent), the patients had a more well-developed left MSG (χ^2^ = 5.53, *p* = 0.019).

The number and degree of development of the insular gyri did not significantly differ between the control groups (24 subjects for BD and 33 for MDD).

### Insular gross anatomy and clinical features

The insular gross anatomy in BD and MDD patients was not associated with IQ, age of onset, the duration of illness, the previous number of affective episodes, or medication at scanning [medicated (*N* = 33) *vs.* non-medicated (*N* = 23) MDD patients, VPA treated (*N* = 12) *vs.* non-VPA treated (*N* = 14) BD patients, and Li treated (*N* = 12) *vs.* non-Li treated (*N* = 14) BD patients] (**Supplementary Tables 1, 2**). Furthermore, the insular gross anatomy was not associated with the MDD subgroups (currently depressed or remitted), psychotic symptoms, or a family history of affective disorders in BD patients, while the rate of comorbid anxiety disorders was higher in MDD patients with a well-developed left AG than in those without (χ^2^ = 4.55, *p* = 0.033). The number of left short insular gyri was higher in MDD patients with anxiety disorders (mean = 3.18 ± 0.73; U = 477.0, *p* = 0.033) compared to those without (mean = 2.76 ± 0.66) (**Supplementary Table 2**).

In the currently depressed MDD subgroup, patients with a well-developed left MSG had a higher BDI score (mean = 39.6 ± 8.9; U = 44.0, *p* = 0.030) and lower MASQ high positive affect score (mean = 41.0 ± 14.3; U=39.5, *p* = 0.039) than in those without (BDI score = 30.8 ± 8.7, MASQ high positive affect score = 50.0 ± 8.9).

In the Melbourne healthy control group, subjects with a left well-developed AG had lower MASQ distress (mean = 23.7 ± 8.2; U = 36.5, *p* = 0.025) and depression (mean = 15.0 ± 4.9; U = 43.0, *p* = 0.043) scores than those without (MASQ distress = mean = 29.1 ± 8.1, MASQ depression = 20.7 ± 7.4).

However, these results of the relationship between the insular gross anatomy and clinical features, including the subgroup analyses, did not survive the Bonferroni correction for multiple comparisons.

### Insular gross anatomy and gray matter volume

In the Melbourne healthy control group (*N* = 33), the gyral number in the left short insula positively correlated with its gray matter volume (rho = 0.419, *p* = 0.015); however, this relationship was no longer significant after Bonferroni corrections. The gray matter volume of the left short insula in the Melbourne healthy control group was larger in those with a well-developed left MSG (*N* = 14, mean = 5858 ± 822 mm^3^) than in those without (*N* = 19, mean = 5200 ± 836 mm^3^) (U = 189.0, *p* = 0.042).

A relationship was not observed between the insular gross anatomy and gray matter volumes in the MDD, BD (**Supplementary Table 1**), or Sydney healthy control groups.

## Discussion

We believe this to be the first report of a more well-developed short insular gyri in BD and MDD patients than in their matched healthy controls using MRI. This gross anatomical feature of affective disorders was more evident in the BD group, but contributed to the severity of anxiety and depressive symptoms in the MDD group. However, the insular gross morphology was not associated with the illness duration or medication status of these patients, supporting its potential role as a stable vulnerability marker of affective disorders.

Gross anatomical variations in the insular cortex, which is predominantly formed during 17 and 35 weeks of gestation along with neural development (7, 9, 35), have also been reported in healthy subjects (10–12); however, their functional significance remains largely unknown. On the other hand, Heschl’s gyrus (HG) duplication, another normal anatomical variation, has been associated with regional brain dysfunction and cognitive deficits (e.g., learning disabilities) in healthy subjects (36, 37). We previously demonstrated that HG duplication increased in various neuropsychiatric disorders, such as schizophrenia spectrum (38–40) and affective (30) disorders, and appeared to be associated with the clinical characteristics (e.g., symptom severity, cognitive deficits) of patients. Collectively, these findings and the present results appear to support the general role of neurodevelopmental abnormalities associated with gyral formation during the embryonic period (8, 9), which may lead to brain dysconnectivity (13), in neuropsychiatric disorders. The regional specificity of brain gyrification patterns in the context of the pathophysiology of neuropsychiatric disorders as well as the specific role of the insular gross morphology need to be examined in multimodal studies on brain function/connectivity.

The present result showing an increased number of short gyri bilaterally in BD patients is consistent with the hypothesis that developmentally mediated neurobiological changes related to socio-emotional neural circuits, including the insular cortex (1, 2), may contribute to the pathophysiology of BD (17, 18). The results obtained herein also suggest a shared macroscopic feature of the insular cortex (i.e., well-developed AG and MSG) between schizophrenia (14–16) and BD. This appears to, at least partially, support the notion of the common neurodevelopmental pathology between these disorders (13, 41), which has been suggested by broad similarities in neurocognitive (42, 43), neuroimaging (44, 45), and genetic (46–48) findings. However, a family history of affective disorders, which implies strong genetic/biological factors, and the comorbid psychotic symptoms of BD patients were not associated with the insular gross anatomy, suggesting its complex contribution to the pathophysiology of BD. Furthermore, the present results and our previous MRI findings (3) indicate that the gross gyral pattern and gray matter reduction in the insular cortex are independent and may reflect the different pathological processes of BD.

Similar to the results observed in BD patients in the present study, MDD patients exhibited a more developed short insular gyri than their matched controls. This result is consistent with previous neuroimaging evidence of shared structural (25) and functional (26) abnormalities in the insular cortex between BD and MDD, supporting aberrant cortical development potentially serving as a vulnerability marker of MDD (49). However, the degree of the change in the insular gyral pattern was less pronounced in the MDD group than in the BD group, which may reflect a less prominent neurodevelopmental pathology and the strong impact of acquired environmental factors in the etiology of MDD (19). We also demonstrated that the well-developed AG and MSG in MDD patients might correlate with comorbid anxiety disorders and the severity of depressive symptoms during active depressive episodes. Therefore, the potential impact of early neurodevelopmental processes during embryonic insular gyral formation on the phenomenology of MDD in later life may involve interactions with environmental factors in epigenetic mechanisms (20, 21). On the other hand, the well-developed AG in healthy control subjects was related to ‘lower’ distress and depression scores, suggesting different mechanisms in the insular structure-function relationship between the pathological status and non-clinical population.

There are several limitations that need to be addressed. First, MRI scans on BD and MDD patients used different settings, which limited the comparability of the results obtained. Therefore, control groups matched for MR settings and the demographic backgrounds of the BD and MDD groups were used in this study. Furthermore, the macroscopic features of the insular cortex did not significantly differ between these independent control groups, supporting the consistency of our methodology. Nevertheless, future studies with complete consistency of MRI settings would enhance the reliability of our findings. Second, the number of participants, especially in the BD group (*N* = 26), was relatively small. Left MSG development significantly differed between MDD patients and healthy controls; however, the present results indicated that the development of both the MSG and AG contributed to anxiety and depressive symptoms in these patients. Therefore, a replication study with a larger MDD cohort may have the capacity to identify differences in the AG developmental pattern from that in controls. In addition, the present study on BD patients in remission enabled us to investigate the potential contribution of the insular gross anatomy to the severity of their symptoms. Thus, a larger sample size with more detailed clinical data (e.g., illness stages, types of depression, and treatment responses) could improve the reliability and generalizability of our findings, particularly in relation to the clinical diversity within affective disorders. Third, while the insular anatomy was related to cognitive function in schizophrenia (16), the present study did not assess cognitive impairment in BD or MDD patients. Our study also did not include direct functional measures such as functional MRI or studies related to functional connectivity. Including such measures could help better understand how the insular structural changes translate into cognitive and emotional dysfunctions in affective disorders. Finally, the structural/functional abnormalities of the insular cortex have also been reported in other neuropsychiatric disorders, such as borderline personality (50, 51) and autism spectrum (52, 53) disorders. Therefore, future research could focus on investigating the specificity of these changes in the context of affective disorders, which would help identify unique anatomical features of BD and MDD.

In summary, our results show that BD patients share the gross brain anatomical feature of an increased number of insular gyri with patients diagnosed with schizophrenia, which may partly underlie the overlap in phenomenology and neurobiology between BD and schizophrenia. Moreover, embryonic neurodevelopmental processes related to gyral formation may have an impact on the severity of symptoms during a later depressive episode in MDD patients. Future multimodal imaging studies on brain function and connectivity are needed to further clarify the potential functional significance and pathological role of the gross anatomy of the insula in neuropsychiatric disorders.

## Data Availability

The raw data supporting the conclusions of this article will be made available by the authors, without undue reservation.
