# Supplementary material for: Gross anatomical features of the insular cortex in affective disorders
Source: Front Psychiatry. 2024 Dec 9;15:1482990. doi: 10.3389/fpsyt.2024.1482990 (PMC11663932; doi:10.3389/fpsyt.2024.1482990)
Supplement: Supplementary file 1 [file DataSheet1.pdf]

Supplementary Table 1. Correlation between the number of short insular gyri and clinical variables and short insular gray matter volumes.

	BD patients (*N* = 26)				Whole MDD patients (*N* = 56)				Currently depressed MDD patients (*N* = 29)				Remitted MDD patients (*N* = 27)			
	Number of short gyri				Number of short gyri				Number of short gyri				Number of short gyri			
	Left		Right		Left		Right		Left		Right		Left		Right	
	rho	*p*	*rho*	*p*	*rho*	*p*	*rho*	*p*	*rho*	*p*	*rho*	*p*	*rho*	*p*	*rho*	*p*
Current IQ	-0.072	0.734	0.220	0.291	-0.167	0.223	0.029	0.833	-	-	-	-	-	-	-	-
Onset age (years)	-0.134	0.514	0.043	0.837	-0.101	0.460	-0.051	0.709	-	-	-	-	-	-	-	-
Duration of illness (years)	0.093	0.650	-0.017	0.935	-0.03	0.825	0.142	0.297	-	-	-	-	-	-	-	-
Number of depressive episodes	0.257	0.204	-0.047	0.821	0.043	0.794	0.304	0.056	-	-	-	-	-	-	-	-
Number of manic episodes	0.391	0.048	-0.022	0.913	-	-	-	-	-	-	-	-	-	-	-	-
Beck Depression Inventory	-	-	-	-	-	-	-	-	0.367	0.05	-0.137	0.479	0.144	0.474	0.107	0.594
PANAS positive affect	-	-	-	-	-	-	-	-	-0.258	0.185	0.135	0.493	-0.175	0.403	-0.212	0.308
PANAS negative affect	-	-	-	-	-	-	-	-	0.153	0.438	-0.039	0.843	0.300	0.136	0.053	0.798
MASQ general distress	-	-	-	-	-	-	-	-	0.249	0.202	0.076	0.702	0.144	0.491	-0.005	0.980
MASQ general depression	-	-	-	-	-	-	-	-	0.035	0.858	-0.349	0.069	0.076	0.719	-0.112	0.594
MASQ general anxiety	-	-	-	-	-	-	-	-	0.142	0.473	0.145	0.461	0.011	0.96	0.202	0.333
MASQ anxious arousal	-	-	-	-	-	-	-	-	0.136	0.490	0.198	0.313	0.154	0.484	0.092	0.677
MASQ high positive affect	-	-	-	-	-	-	-	-	-0.383	0.044	0.231	0.236	0.151	0.481	0.057	0.790
MASQ loss of interest	-	-	-	-	-	-	-	-	0.071	0.719	-0.326	0.091	0.041	0.847	-0.023	0.913
Left short insular volume	-0.133	0.516	-	-	0.110	0.420	-	-	-	-	-	-	-	-	-	-
Right short insular volume	-	-	0.044	0.831	-	-	0.136	0.317	-	-	-	-	-	-	-	-

BD, bipolar disorder; MASQ, Mood and Anxiety Symptom Questionnaire; MDD, major depressive disorder; PANAS, Positive and Negative Affect Schedule. Symptom ratings were separately investigated for currently depressed and remitted MDD subgroups.

Supplementary Table 2. Mann-Whitney U test results of the relationship between the number of short insular gyri and clinical subgroups.

Clinical subgroups	Number of left short gyri		Number of right short gyri	
	U	*p*	U	*p*
Currently depressed or remitted MDD subgroups	339.0	0.351	415.5	0.667
Comorbid anxiety disorders in the MDD group	477.0	0.033	409.5	0.380
Medicated or non-medicated MDD subgroups	394.0	0.097	345.5	0.512
History of psychosis in the BD group	75.0	0.816	71.0	0.660
Family history of affective disorders in the BD group	73.0	0.737	91.0	0.586
VPA treated or non-VPA treated BD patients	83.5	0.980	62.0	0.274
Li treated or non-Li treated BD patients	65.0	0.347	72.0	0.560

BD, bipolar disorder; Li, lithium; MDD, major depressive disorder; VPA, valproate.
